# Development of Optimized Inhibitor RNAs Allowing Multisite-Targeting of the HCV Genome

**DOI:** 10.3390/molecules22050861

**Published:** 2017-05-22

**Authors:** Cristina Romero-López, Thomas Lahlali, Beatriz Berzal-Herranz, Alfredo Berzal-Herranz

**Affiliations:** Instituto de Parasitología y Biomedicina “López-Neyra”, IPBLN-CSIC, PTS Granada, Av. del Conocimiento 17, Armilla, 18016 Granada, Spain; thomas.lahlali@inserm.fr (T.L.); bbh@ipb.csic.es (B.B.-H.)

**Keywords:** RNA aptamer, hepatitis C virus, IRES, RNA targeting

## Abstract

Engineered multivalent drugs are promising candidates for fighting infection by highly variable viruses, such as HCV. The combination into a single molecule of more than one inhibitory domain, each with its own target specificity and even a different mechanism of action, results in drugs with potentially enhanced therapeutic properties. In the present work, the anti-HCV chimeric inhibitor RNA HH363-10, which has a hammerhead catalytic domain and an aptamer RNA domain, was subjected to an in vitro selection strategy to isolate ten different optimised chimeric inhibitor RNAs. The catalytic domain was preserved while the aptamer RNA domain was evolved to contain two binding sites, one mapping to the highly conserved IIIf domain of the HCV genome’s internal ribosome entry site (IRES), and the other either to IRES domain IV (which contains the translation start codon) or the essential linker region between domains I and II. These chimeric molecules efficiently and specifically interfered with HCV IRES-dependent translation in vitro (with IC_50_ values in the low µM range). They also inhibited both viral translation and replication in cell culture. These findings highlight the feasibility of using in vitro selection strategies for obtaining improved RNA molecules with potential clinical applications.

## 1. Introduction

The ability to interfere at the genetic level with the functioning of RNA viruses has long been an area of interest. One of the most promising lines of research in this respect has involved the development of nucleic acid-based inhibitory molecules. Therapeutic nucleic acids have advantages over other targeted drugs such as those based on antibodies; for example, they show low immunogenicity and their production is easier, minimizing structural variations among different batches. However, their low biostability, a consequence of their rapid renal filtration and degradation by nucleases, is a problem. Fortunately they can be modified to avoid these drawbacks, for example by adding groups that improve either their uptake by cells, their binding affinity, or their stability against nucleases [1].

The use of aptamers as therapeutic and diagnostic agents has become a feasible option. Aptamers are single-stranded DNA or RNA molecules that bind in a very specific and efficient manner to their targets. Aptamers are isolated by an in vitro selection procedure known as SELEX (systematic evolution of ligands by exponential enrichment) [2,3]. From a highly heterogeneous initial population, usually composed of 10^12^ to 10^15^ variants, consecutive selection rounds identify aptamers against specific targets. The fact that the aptamer action can be reversed by an antidote has drawn them much clinical interest [4,5,6].

Strong target affinity is critical in the development of an efficient aptamer. Numerous authors ([7,8] and references therein) report this to have been achieved using the SELEX process by modifying the temperature or ionic conditions. The incorporation of chemical modifications, either during the in vitro selection procedure or as post-SELEX editing process, also allows the optimization of the aptamers available [9,10,11,12]. The incorporation of further anchoring sites, yielding multivalent molecules, can also increase the affinity of monovalent compounds [13,14,15,16,17]. A conventional way of designing multivalent aptamers is to connect independent modules. The main drawback of this strategy is the need to preserve aptamer folding. In addition, the concatenation of multiple monomers produces long molecules that are hard to synthesize and deliver into cells.

**Figure 1:**
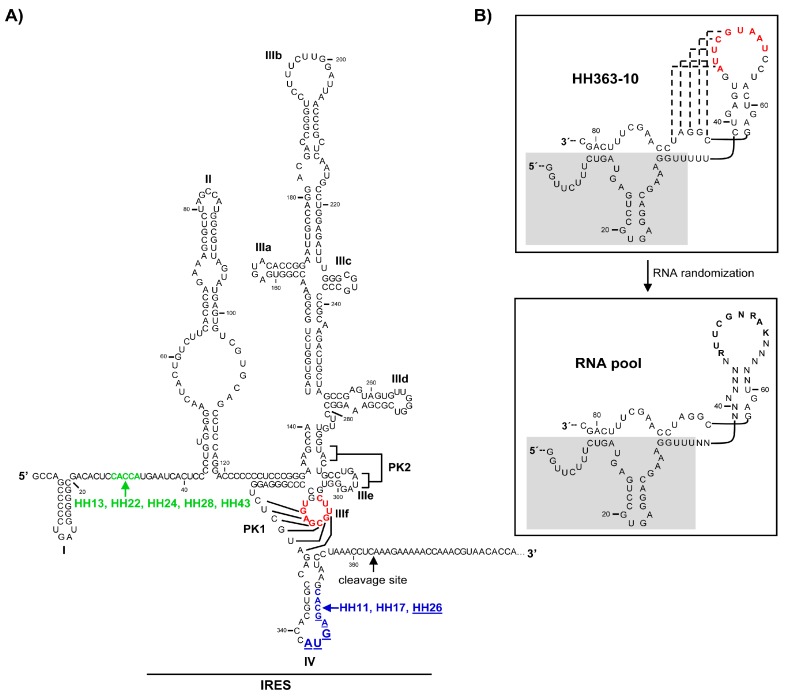
The HCV IRES region and the parental HH363-10 chimeric inhibitor employed as the prototype for the construction of the RNA pool. (**A**) Sequence and secondary structure of the HCV IRES including the functional RNA domains targeted by the selected chimeric inhibitory RNA molecules. The IRES site cleaved by HH363-10 is indicated by an arrow. The translation start codon at position 342 is enlarged. The nucleotides that interact with the aptamer domain of HH363-10 are shown in red. Residues proposed to interact with the aptamer domain of HH-11 and HH-17 are shown in blue. The theoretical anchoring site for HH-26 is indicated in blue and underlined. Nucleotides pictured in green, located in the linker region between domains I and II, likely act as a binding region for the aptamer domain of the chimeric inhibitors HH-13, HH-22, HH-24, HH-28 and HH-43; (**B**) Sequence and theoretical secondary structure model of HH363-10. Figure was adapted from [14]. The catalytic domain, HH363, is shadowed. Tertiary contacts are indicated by dotted lines. Residues in the aptamer domain responsible for the interaction with domain IIIf of the IRES are shown in red. Randomization of the residues flanking this sequence motif, plus partial mutagenesis of those nucleotides participating in the IIIf binding site, yielded an initial population of more than 6 × 10^7^ theoretical variants (lower panel). R, G or A; K, G or U; PK, pseudoknot.

Our group previously isolated the RNA inhibitor HH363-10 using an innovative in vitro selection strategy [13,14]. This method uses two sequential steps of selection for two distinct activities, binding to the HCV IRES region and cleavage at nucleotide 363 of the genomic viral RNA. This procedure yielded chimeric RNA molecules composed of two inhibitory RNA domains, an aptamer and a catalytic domain. HH363-10 can bind to the highly conserved IIIf domain of the essential internal ribosome entry site (IRES) in the HCV genome through the aptamer domain (Figure 1) [13,14]. Domain IIIf is a key element in the three-dimensional organization of the IRES, and participates extensively in the recruitment and assembly of the 40S ribosomal particle [18,19]. It therefore appears as an interesting candidate target for the development of new antiviral molecules. HH363-10 also harbours a hammerhead ribozyme domain that cleaves (though at low efficiency) the viral RNA at a position spatially close to the IIIf aptamer-targeting site (Figure 1A). This design provided a potent inhibitory RNA able to interfere with HCV translation in cell culture [14]—a prototype molecule for further improvement.

The present work reports the isolation of novel chimeric inhibitory RNA molecules based on HH363-10. These bear the parental anchoring site for the domain IIIf in the aptamer component, but also possess a secondary anchoring site that does not interfere with the catalytic properties of the hammerhead component. The resulting molecules efficiently interfered with HCV IRES-dependent translation in vitro and in a human-derived hepatoma cell line. Further, they strongly inhibited viral RNA synthesis, confirming the potential of multivalent RNA molecules as therapeutic agents for use against HCV.

## 2. Results

### 2.1. Isolation of Improved RNA Molecules that Interfere with HCV IRES Function In Vitro

**Figure 2:**
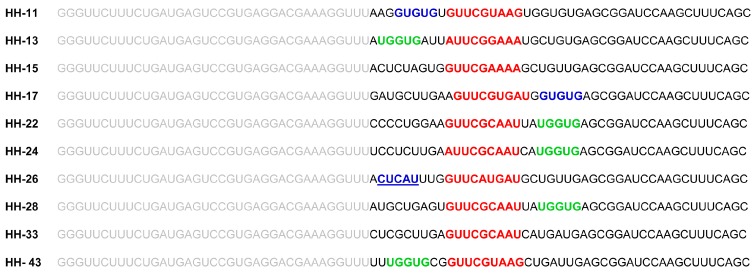
Selected RNA molecules targeting the HCV IRES region. Sequences of the 10 selected chimeric inhibitory RNAs after seven rounds of selection. Residues in grey denote the conserved catalytic domain of the inhibitory RNA. The sequence motif involved in the interaction with the domain IIIf is indicated in red. The nucleotides theoretically targeting domain IV and the linker sequence between domains I and II within the IRES are pictured in blue and green respectively. The unique sequence in HH-26 that binds to the apical loop of IRES domain IV is also underlined.

The chimeric inhibitor HH363-10 was subjected to the previously described in vitro selection procedure [13,14], with the aim of identifying inhibitory RNA molecules that act against the IRES region of the HCV genome, and that showed improved aptamer and catalytic properties. For this, an RNA pool was designed (Figure 1B), based on the HH363-10 inhibitor, adhering to the following criteria: (i) the maintenance of the sequence motif responsible for the aptamer’s interaction with the domain IIIf of the IRES; (ii) randomization of the nucleotides flanking the interacting sequence with the aim of isolating RNA molecules bearing secondary anchoring sites at the HCV IRES region besides the primary domain IIIf. This RNA pool was then subjected to in vitro selection as previously described [13]. After seven selection rounds, 10 RNA variants (HH-11, HH-13, HH-15, HH-17, HH-22, HH-24, HH-26, HH-28, HH-33 and HH-43; Figure 2) were isolated and analysed for their ability to inhibit IRES-dependent translation in vitro. Sequence analysis using the RNAup software tool [20] showed new theoretical interaction sites to map to the conserved domain IV, and to the interdomain I-II regions (Figure 1A). This showed the selection strategy to have worked properly.

**Figure 3:**
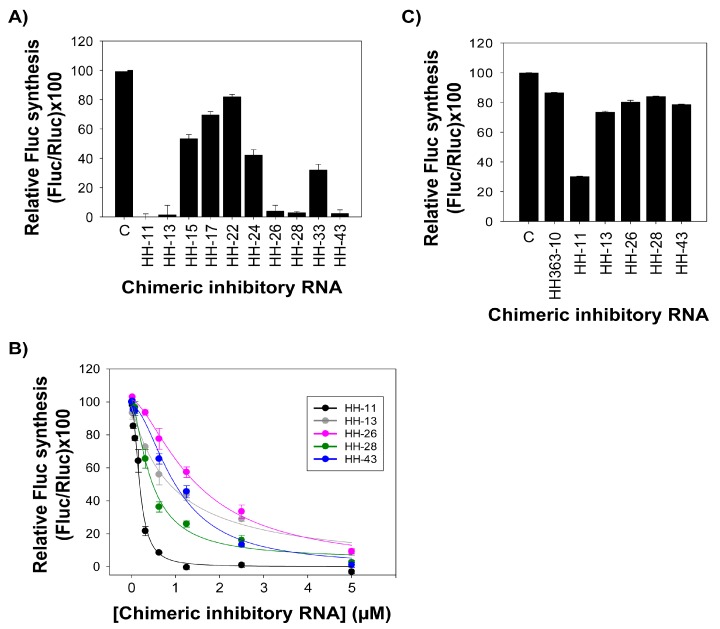
Specific inhibition of HCV IRES-dependent translation in vitro by the selected chimeric inhibitory RNAs. (**A**) The bar chart shows the relative synthesis of FLuc protein directed by the HCV IRES region achieved in the presence of 5 µM of each chimeric inhibitory RNA, normalized to that obtained for the cap-dependent translation of cap-RLuc mRNA. The resulting values were referred to the results obtained in the absence of inhibitory RNA. Data are the mean of at least three independent assays ± standard deviation represented by the error bars; (**B**) The plot shows the relative reduction in FLuc synthesis caused by the chimeric inhibitors HH-11, HH-13, HH-26, HH-28 and HH-43. Data were normalized to those obtained for the synthesis of the RLuc reporter protein. The relative amount of FLuc obtained at each inhibitor concentration is calculated as a percentage with respect to the control reaction in the absence of any chimeric inhibitory RNA. Data (the mean of three independent assays ± standard deviation) were fitted to a non-linear regression curve to determine the IC_50_ values; (**C**) The histogram shows the effect of each anti-HCV inhibitory RNA on the IRES function of the closely related GBV-B virus. The synthesis of the FLuc protein was normalized as noted in A). Data are the mean of three independent assays ± standard deviation.

The activity of the different chimeric inhibitors was examined in in vitro translation assays involving two monocistronic RNA molecules (used as templates) in rabbit reticulocyte lysates [14]. One of these monocistronic RNAs, IRES-FLuc, encompasses the HCV IRES region directing the synthesis of the reporter FLuc protein; the other, cap-RLuc, codes for the RLuc enzyme (which is translated in a cap-dependent manner). Each inhibitor was assayed at a concentration of 5 µM. HH-11, HH-13, HH-26, HH-28 and HH-43 efficiently interfered with IRES function (Figure 3), showing inhibition values close to 97%. HH-15, HH-17, HH-22, HH-24 and HH-33 also promoted a reduction in FLuc synthesis, but to a lesser extent (20–60%). Cap-dependent translation was not affected (<5%, data not shown) for any of the assayed chimeric inhibitory RNAs at the concentration tested, demonstrating the specific nature of the inhibition of IRES function.

The potential of the five most efficient chimeric inhibitor RNAs—HH-11, HH-13, HH-26, HH-28 and HH-43—as IRES interfering agents was then further characterised using different concentrations of each, ranging from an IRES-FLuc:inhibitor RNA molar ratio of 1:1 to 8:1. Potent dose-dependent inhibition was recorded for HH-11, with an IC_50_ value of 170 ± 20 nM and a maximum inhibition of close to 100% (Table 1; Figure 3B). These results are better than those reported with the parental chimeric inhibitor HH363-10 [14], which showed a maximum inhibition of 90%. More importantly, maximum inhibition with HH-11 was attained at 1 µM, whereas HH363-10 required a concentration of 2.5 µM to achieve the 90% inhibition. Inhibitor RNAs HH-13, HH-26, HH-28 and HH-43 also affected the function of the HCV IRES in a dose-dependent manner, with IC_50_ values in the low µM range and maximum inhibition values of 90–95% (Table 1; Figure 3B).

To further investigate the specificity of the inhibitors against the HCV IRES region, in vitro translation assays were performed with the construct IRES_GBV-B_ -FLuc, which bears the IRES region of the closely related hepacivirus GBV-B [21] fused to the coding sequence for the FLuc protein [15]. Interestingly, only the chimeric inhibitor HH-11 significantly affected GBV-B IRES activity (Figure 3C), while the rest of the tested inhibitory RNAs showed marked specificity for the HCV IRES region. The notable conservation of the 5’UTR among hepacivirus suggests that HH-11 might target a common key structural motif, thus resulting in the inhibition of GBV-B IRES activity. Other sequence motifs in the aptamer domain might also target the GBV-B IRES. Finally, it should be noted that although HH-11 interfered with GBV-B IRES-dependent translation, it did not affect cap-mediated RLuc synthesis (data not shown). This shows that HH-11 is not a generic translation inhibitor, but rather that it is specific for sequence and/or structural motifs in the hepacivirus IRES.

Taken together, these results show that the isolated chimeric inhibitor RNAs operate as improved inhibitors of HCV IRES function compared to the prototype HH363-10.

### 2.2. The Chimeric Inhibitor RNAs Inhibit HCV IRES-Dependent Translation in Cell Culture

**Figure 4:**
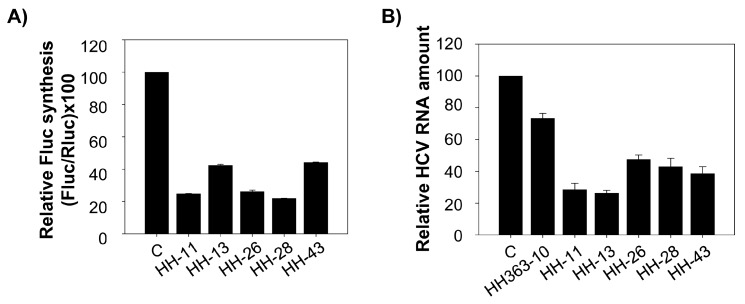
The selected chimeric inhibitory RNAs interfere with HCV translation and replication in a human hepatoma cell line. (**A**) Huh-7 cells were transfected with 5 µg of each chimeric inhibitory RNA or the non-related RNA80, plus 1.1 µg of a mixture containing the IRES-FLuc mRNA and the cap-RLuc. Luciferase activity was measured 18 h after transfection and the values turned into percentages by reference to the results obtained in the control assay with RNA80. Data are the mean of four independent experiments ± standard deviation represented by the error bars; (**B**) Five µg of the different chimeric inhibitory RNAs were used to transfect Huh-7 cells stably supporting HCV replication. Total RNA was extracted 24 h post-transfection using TRIzol™, and the relative amount of HCV RNA quantified by RT-qPCR. Data are the mean of four independent assays ± standard deviation represented by the error bars.

The effect of the HH-11, HH-13, HH-26, HH-28 and HH-43 chimeric inhibitors on HCV IRES-dependent translation was also evaluated in cell culture. Human hepatoma cell line Huh-7 was co-transfected with a mixture containing the RNA constructs IRES-FLuc and cap-RLuc and a molar excess of an inhibitory RNA (25:1). The resulting data were compared with those obtained in the presence of a non-related RNA molecule, RNA80 [14,16], used to complement the total RNA amount in the transfection mix. The five tested molecules provoked a significant reduction (60–80%) in HCV-IRES activity (Figure 4A). HH-11 appeared as a potent inhibitory RNA, in good agreement with the results of the in vitro translation assays (Figure 3A). HH-26 and HH-28 also induced significant reductions (nearly 80%) in IRES activity, showing them to be improved anti-HCV agents.

### 2.3. Interference with HCV Replication by the Chimeric Inhibitors

The effect of HH-11, HH-13, HH-26, HH-28 and HH-43 on HCV replication was also investigated. Huh-7 cells bearing a subgenomic replicative RNA molecule derived from HCV (Huh-7 NS3-3’; [22,23]) were transiently transfected with 175 nM of inhibitory RNA or the non-related RNA80 molecule. The amount of replicative HCV RNA was determined 24 h post-transfection by RT-qPCR, and normalized to that of the intracellular mRNA encoding for GAPDH protein (see Materials and Methods). The selected chimeric inhibitors significantly interfered with HCV replication (Figure 4B), promoting a 60–80% reduction in viral RNA positive strands with respect to the control assay. Interestingly, these data contrast with those obtained for HH363-10 (Figure 4B), which affected HCV replication much more weakly (~25% reduction in HCV RNA levels). This result confirms the improved inhibitory ability of the selected chimeric inhibitor RNAs over the parental HH363-10.

## 3. Discussion

The development of novel diagnostic and therapeutic tools for dealing with viral infections is a major scientific goal. In recent years, aptamers have become candidates as therapeutic alternatives to monoclonal antibodies, which are highly immunogenic and expensive to produce. Aptamers can also be optimized to provide even more efficient inhibitors, as long as their three dimensional structure is preserved. Many strategies are now available to improve the affinity of aptamers for their targets, to increase their resistance to nucleases, and to improve their delivery to cells ([24] and references therein).

Conventional monovalent aptamers have short retention times at their targets, reducing their therapeutic indices, but multivalent aptamers, bearing at least two different targeting sites, demonstrate improved affinity [25]. Multivalent aptamers are usually produced by the consecutive joining of monovalent monomers, either directly or via linkers. The main drawback here is that the structure of the aptamer can be altered, which can reduce its clinical effectiveness. In addition, the increase in the aptamer length results in a more expensive and less efficient production. Customized in vitro selection strategies, however, allow new targeting sites to be added to an inhibitory molecule’s repertoire without disturbing its conformation.

The chimeric RNA molecule HH363-10 has both catalytic and aptamer properties, and targets two sites in the HCV IRES region [14]. In cell culture, it can reduce viral translation by ~50%. In the present work, in vitro selection was used to further improve its inhibitory properties. Ten variants were isolated, all of which were able to interfere with HCV IRES function (Figure 2 and Figure 3). HH-11, HH-13, HH-26, HH-28 and HH-43 induced a strong reduction in viral translation both in vitro and in cell culture, with IC_50_ values in the low µM range achieved (Figure 3B), and inhibition values up to almost 100% obtained (Figure 3B). The properties of the parental compound HH363-10 were therefore improved [14].

**Figure 5:**
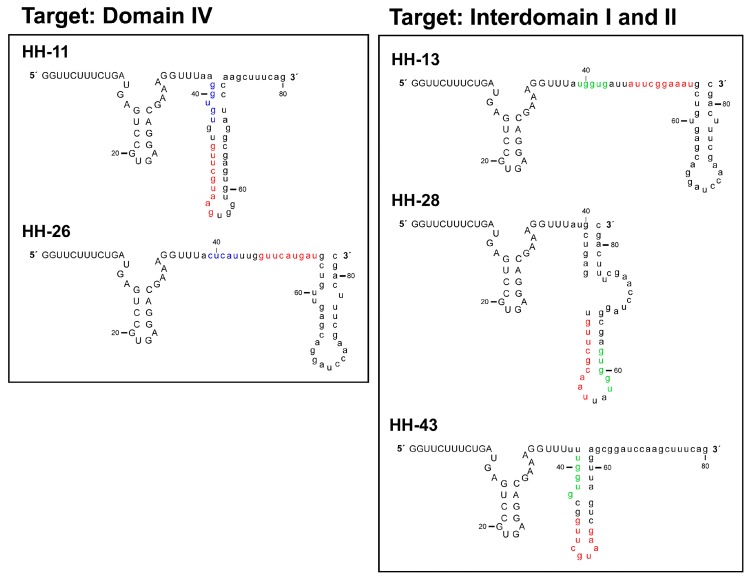
Theoretical model for the folding of the chimeric inhibitory RNAs HH-11, HH-13, HH-26, HH-28 and HH-43. In silico studies using the RNAstructure software [26] were performed to model the secondary structure of the RNA molecules under study; the figure shows the results obtained. The colour code is the same as that indicated in Figure 2.

RNAup [20] and RNAstructure [26] software have been used in in silico searches for target sites within the IRES beyond the original anchoring region for the aptamer in the domain IIIf. The presence of additional anchoring sites in the chimeric inhibitor RNAs might explain the improvement in the inhibitory capacity of the novel selected chimeric ones (Figure 5). The present analyses revealed HH-11 and HH-26 to have acquired an additional anchoring region with a target in the 3’ flank and the apical loop of IRES domain IV respectively (Figure 1, Figure 2 and Figure 5). Domain IV is close to the primary target site in domain IIIf, suggesting that a single inhibitor RNA might bind to both. However, it cannot be ruled out that two different molecules of the same inhibitor use their different anchoring sites in a shared attack on different IRES sites. The identified sequence motifs of 5′-GUGUG-3′ for HH-11, and 5′-CUCAU-3′ for HH-26, consist of five residues upstream of the parental anchoring sequence (which has 9 nt) (Figure 1, Figure 2 and Figure 5). It is tempting to speculate that the first interaction between inhibitor and target is directed by the longer primary motif targeting the domain IIIf, with the second site then helping to stabilize the resulting complex. It is interesting that intact domain IV conformation and stability are required for efficient IRES-dependent translation [27]. This might help explain the potent inhibitory activity of the chimeric inhibitor RNAs HH-11 and HH-26, which target this domain.

The molecules HH-13, HH-28 and HH-43 were predicted to bear a sequence motif in their aptamer domain, 5′-UGGUG-3′, able to interact with the linker region between domains I and II (Figure 1, Figure 2 and Figure 5). This contact can be used as a secondary anchorage after the initial interaction with domain IIIf. The linker sequence in the IRES might locate spatially close to the pseudoknot structure by virtue of a long range RNA-RNA interaction with domain VI [28], which fits well with the hypothesis that both anchoring sites could be used by a single, multivalent compound. It has also been demonstrated that this region is the target of the essential microRNA miR-122. This interaction promotes both viral translation and replication [29,30,31]. This agrees well with the extensive inhibitory effect promoted by the chimeric inhibitory RNAs.

It should be noted that HH-17 shares the second interacting sequence, 5′-GUGUG-3′, with HH-11, while HH-22 and HH-24 acquired the sequence motif 5′-UGGUG-3′, which is present in the chimeric inhibitors HH-13, HH-28 and HH-43 (Figure 1 and Figure 2). Interestingly, they only showed slight inhibitory activity, suggesting that factors beyond the incorporation of new anchoring sequence motifs are involved in anti-HCV activity, e.g., the aptamer structure.

In conclusion, the present work shows that in vitro selection allows chimeric inhibitory RNAs to be obtained that target three different sites in the HCV genome. These inhibitors might be further optimized by the introduction of nucleotide analogues and/or chemically modified nucleotides to provide efficient antiviral drugs. This strategy should be extended to other viral diseases in order to develop further sets of molecules with potent therapeutic properties.

## 4. Materials and Methods

### 4.1. Cell Lines and Culture Conditions

Cell monolayers of the human hepatoma cell line Huh-7 were maintained in Dulbecco’s modified Eagle medium (DMEM) supplemented with 10% heat-inactivated foetal bovine serum (FBS; Invitrogen™, Waltham, MA, USA) and 1 mM sodium pyruvate (Sigma, St. Louis, MO, USA), at 37 °C in a 5% CO_2_ atmosphere. 

Huh-7-NS3-3′ cells supporting an HCV subgenomic replicon (genotype 1b) were grown in DMEM supplemented with 20% heat-inactivated FBS, 1 mM sodium pyruvate (Sigma) and 0.5 mg/mL G-418. The subgenomic replicon construct consists of the HCV-IRES (genotype 1b), followed by the neomycin phosphotransferase gene, the IRES region of the encephalomyocarditis virus (EMCV), the coding sequence for non-structural HCV proteins (NS3–NS5), and the HCV 3’UTR.

### 4.2. DNA Templates and RNA Synthesis

DNA templates encoding the chimeric inhibitors HH363-10, HH-11, HH-13, HH-15, HH-17, HH-24, HH-26, HH-28, HH-33 and HH-43 were obtained by PCR as previously described [13]. The coding sequence for GB virus B (GBV-B), IRES_GBV-B_-FLuc, was obtained by PCR amplification as previously reported [16]. The RNA constructs IRES-FLuc, cap-RLuc and RNA80 were synthesized as previously described [14,16].

RNA molecules were obtained by in vitro transcription using the RiboMAX™-T7 large-scale RNA production system (Promega, Madison, WI, USA) following the manufacturer’s instructions. During the synthesis of cap-RLuc, a cap structure was incorporated into its 5’ end by adding 2 mM of Ribo m^7^G Cap analog (Promega). The resulting RNAs were purified by phenol/chloroform extraction and size exclusion chromatography (Sephadex G-25; GE Healthcare, Little Chanfont, UK). The amount and quality of the resulting RNA samples were determined by A_260_ and A_280_ measurements, followed by agarose-formaldehyde gel electrophoresis.

### 4.3. In Vitro Translation Assays

IRES-FLuc and cap-RLuc mRNAs were in vitro translated using the Flexi^®^ rabbit reticulocyte lysate system (Promega). Reactions proceeded in 6 µL volumes containing 4 µL cell extract, 1 mM of an amino acid mixture lacking methionine, 100 mM KCl and 1.5 µCi of a mixture of L-[^35^S]methionine and L-[^35^S]cysteine (all from the Redivue Pro-mix L-[^35^S] in vitro cell labelling mix; GE Healthcare). The RNA templates for the synthesis of FLuc (HCV IRES-FLuc or IRES_GBV-B_ -FLuc) and RLuc (cap-RLuc) proteins were added to a final concentration of 30 ng/µL (~40 nM) and 20 ng/µL (~60 nM) respectively. Inhibitory RNA concentrations ranging from 40 nM to 5 µM were assayed. Prior to their incorporation into the translation mix, all the RNA molecules were denatured by heating at 95 °C for 2 min and cooling at 4 °C for 15 min. Translation proceeded at 30 °C for 60 min. Reactions were stopped by cooling on ice and the protein products resolved on 12.5% (*w*/*v*) denaturing polyacrylamide gels. Dried gels were scanned in a Phosphorimager (Storm 820, GE Healthcare) and quantified using Image Quant 5.2^®^ software (GE Healthcare). The IC_50_ values were calculated using SigmaPlot 8.02^®^ software (Systat Software Inc., San José, CA, USA) from the equation

y = y_max_/(1 + 10^(LogIC^_50_^−X)^)

where y_max_ is the maximum percentage of FLuc relative synthesis, IC_50_ the inhibitor concentration that produces 50% of the maximum observed effect, and X the inhibitor concentration.

### 4.4. RNA Transfection

The cell lines Huh-7 and Huh-7 NS3-3′ were transfected as previously reported [14,15]. To assess the inhibitory activity of the chimeric RNA molecules ex vivo on HCV IRES-dependent translation, Huh-7 cells were used as model system. 90,000 cells/well were seeded onto a 24-well plate and allowed to reach 80–90% confluence (36–48 h). A mix containing 1 µg of the RNA construct IRES-FLuc and 100 ng of cap-RLuc was supplemented with 5 µg of each chimeric inhibitor or the non-related RNA80. TransFectin™ (Bio-Rad, Hercules, CA, USA) was used as the transfection reagent following the manufacturer’s instructions. Luciferase activity was detected 18 h after transfection using the Dual-Luciferase™ reporter assay system (Promega).

Analysis of the inhibitory effect on HCV replication was performed by transfecting Huh-7-NS3-3′ cells with the different chimeric inhibitors under study. At 24 h before transfection, 90,000 cells were seeded and grown until 80% confluence in 1.5 cm diameter dishes in the presence of culture medium without G-418. The cells were then transfected with 4 µg of each inhibitor RNA or RNA80 using TransFectin™ (Bio-Rad). At 24 h post-transfection, the cells were harvested and processed for further RNA extraction.

### 4.5. Relative Quantification of HCV Subgenomic RNA

Intracellular HCV replicon positive-strand RNA levels were measured as previously described [32]. Briefly, total RNA was extracted using TRIzol™ (Invitrogen™ via Thermo Fisher Scientific, Carlsbad, CA, USA) following the manufacturer’s instructions. 50 ng of the purified RNA were then reverse transcribed using the High-Capacity cDNA Reverse Transcription Kit (Applied Biosystems via Thermo Fisher Scientific) and 1 µg of random primers. A fraction of the cDNA product was used for quantitative PCR using the SsoFast™ Evagreen^®^ Supermix Kit (Bio-Rad) and amplified over 40 cycles using specific oligonucleotides targeting the IRES region (C-149 and C-342) [33]. In parallel, RT-qPCR of the mRNA encoding the human glyceraldehyde-3-phosphate dehydrogenase (hGAPDH) was performed using the primers hGAPDH_Fw and hGAPDH_Rev [34]. The resulting data were used to calculate the relative amount of HCV subgenomic RNA. Reactions were performed using the Bio-Rad CFX 96 real time system, and data analyzed using Bio-Rad CFX real time manager software.

## Figures and Tables

**Table 1:** IC_50_ values of the different inhibitory RNAs.

Inhibitor	IC_50_ (µM) ^a^	Relative Fluc Synthesis (%) ^b^
HH363-10 [14]	0.15 ± 0.04	11.22 ± 2.77
HH-11	0.17 ± 0.02	0.01 ± 1.10
HH-13	0.91 ± 0.31	8.78 ± 2.21
HH-26	1.46 ± 0.24	9.25 ± 1.47
HH-28	0.44 ± 0.06	2.56 ± 1.34
HH-43	1.02 ± 0.15	1.00 ± 0.40

^a^ IC_50_ values were derived from the equation y = 100/(1 + 10^(LogIC^_50_^−X)^); ^b^ Data correspond to the highest concentration of inhibitor tested. Values are the mean of three independent assays ± standard deviation.
